# Optimal Control-Based Inverse Determination of Electrode Distribution for Electroosmotic Micromixer

**DOI:** 10.3390/mi8080247

**Published:** 2017-08-11

**Authors:** Yuan Ji, Yongbo Deng, Zhenyu Liu, Teng Zhou, Yihui Wu, Shizhi Qian

**Affiliations:** 1 State Key Laboratory of Applied Optics, Changchun Institute of Optics, Fine Mechanics and Physics (CIOMP), Chinese Academy of Sciences, Changchun 130033, China; jy.93@163.com (Y.J.); yihuiwu@ciomp.ac.cn (Y.W.); 2 University of Chinese Academy of Sciences, Beijing 100039, China; 3 Changchun Institute of Optics, Fine Mechanics and Physics (CIOMP), Chinese Academy of Sciences, Changchun 130033, China; liuzy@ciomp.ac.cn; 4 Mechanical and Electrical Engineering College, Hainan University, Haikou 570228, China; zhou.teng@live.com; 5 Department of Mechanical and Aerospace Engineering, Old Dominion University, ECSB 1309, 4700 Elkhorn Ave, Norfolk, VA 23529, USA; sqian@odu.edu

**Keywords:** electroosmotic micromixer, electrode distribution, optimal control

## Abstract

This paper presents an optimal control-based inverse method used to determine the distribution of the electrodes for the electroosmotic micromixers with external driven flow from the inlet. Based on the optimal control method, one Dirichlet boundary control problem is constructed to inversely find the optimal distribution of the electrodes on the sidewalls of electroosmotic micromixers and achieve the acceptable mixing performance. After solving the boundary control problem, results are also provided to demonstrate the effectiveness of the proposed method; the step-shaped distribution of the external electric potential imposed on the sidewalls is obtained, and the electrodes with an interlaced arrangement are inversely derived according to the obtained external electric potential.

## 1. Introduction

Lab-on-a-chip is the generic term for the integration of microdevices to carry out conventional analytical laboratory tests. Such devices offer significant benefits over traditional laboratory tests in terms of device size as well as sample/reagent usage, and can provide much faster results for chemical and biochemical analyses [1,2]. Because of these advantages, such devices are considered to be a promising option for miniaturization in the area of the environmental and defense monitoring, chemical synthesis, and biomedical applications. Lab-on-a-chip integrates various subcomponents, such as pumps, mixers, reactors, and dilution chambers. Therefore, the study of fluid flow in microscale (i.e., microfluidics) has become central to the development of corresponding devices [3,4,5]. Micromixers are often vital components for lab-on-a-chip devices, as mixing is required for chemical applications, biological applications, and the detection/analysis of chemical or biochemical content [6,7,8].

Owing to small channel dimensions and low flow rates, the Reynolds number of the flows in microfluidic devices is typically very small. Mixing through turbulent flow induced by inertial/viscous effects for aqueous solutions become unfeasible, because diffusion is the dominant mechanism in micromixing due to the absence of turbulence. An effective mixing in low Reynolds number flow regimes can be obtained by the chaotic advection mechanism, which can occur in regular smooth flows [9] and provides an effective increase of the interfacial contact area [10]. Electroosmosis is one of the most common nonmechanical means for achieving chaotic advection in microfluidics. When a charged solid surface comes in contact with an electrolyte, an electric double layer (EDL) of ions is formed due to the interplay between electrical and diffusive forces [11]. The flow of liquids containing dissolved ions under the influence of electrical body forces is known as electroosmosis; it is a subject treated in the electrokinetic transport literature [12,13,14]. Several methods have been systematically discussed for mixing low Reynolds number electroosmotic flows with patterned grooves [15,16]. These grooves induce spiral circulations around the flow axis, and stretch and fold the streams, with the result that a complete mixing is achieved within a short mixing length. The use of unstable electrokinetic flow to achieve a chaotic mixing effect has also been presented in [17,18,19,20,21]. Several numerical analytical investigations on electroosmotic mixing have been performed [13,22,23], and the mixing efficiency has been enhanced based on the periodic electroosmotic flow [24], modulation of electric fields [25,26,27], and shape optimization [28]. The pattern of electroosmotic flow is mainly determined by the electrode distribution. Due to the complexity of electroosmotic flow, physical intuition-based determination of electrode distribution has its limitations. To overcome this limitation, it is necessary to develop the inverse termination method for the electrode distribution of electroosmotic micromixers.

In electroosmotic micromixers, the mixing efficiency is mainly determined by the electrode distribution used to carry the externally applied electric potential. Therefore, this paper is focused on the method used to inversely determine the electrode distribution for electroosmotic micromixers. The discussed inverse termination method is built based on the optimal control method, which has been utilized to implement airfoil design, sensor deployment, and control the convection diffusion and electric field for electrorheological fluids [29,30]. Based on the optimal control method, one boundary control problem is constructed for the electroosmotic micromixer in this paper. After solving the problem, the electrode distribution can be determined according to the obtained step-shaped externally applied electric potential.

## 2. Methodology

### 2.1. Modeling

When a micromixer is used to mix two fluidic flows with different solutes, the desired effect is the mixing of the two flows with anticipated concentration distribution at the outlet of the micromixer. The anticipated concentration distribution at the outlet can be specified by the designer based on the desired performance of the micromixer. The mixing performance of the micromixer can be measured by the least square variance between the obtained concentration *c* and the anticipated concentration $c_{a}$ at the outlet, named “mixing measurement” [8,31]:

(1) $$\begin{array}{c} \Psi \left(c\right)=\int_{\Gamma_{o}}{\left(c-c_{a}\right)}^{2}\;d\Gamma /\int_{\Gamma_{i}}{\left(c_{r}-c_{a}\right)}^{2}\;d\Gamma \end{array}$$

where $\Gamma_{i}$ and $\Gamma_{o}$ are the inlet and outlet of the micromixer, respectively; $c_{r}$ is the reference concentration distribution, which is usually chosen to be the given concentration distribution at the inlet. The required performance for micromixers is that sufficient mixing of the two solutes is achieved. Therefore, the anticipated concentration $c_{a}$ is specified to be the ideal concentration distribution of the solute at the outlet after sufficient mixing. In an electroosmotic micromixer with fixed geometry, the mixing efficiency is determined by the distribution of the external electric potential induced by the electrode potential. The distribution of the electrode potential lies on the distribution of the electrodes at the sidewalls of the electroosmotic micromixer. Then, the problem is how to find a reasonable distribution of the electrodes that minimizes the mixing measurement and achieves sufficient mixing in an electroosmotic micromixer. In this paper, the optimal control method is adopted, and one Dirichelet boundary control problem is constructed to solve this problem.

**Figure 1:**
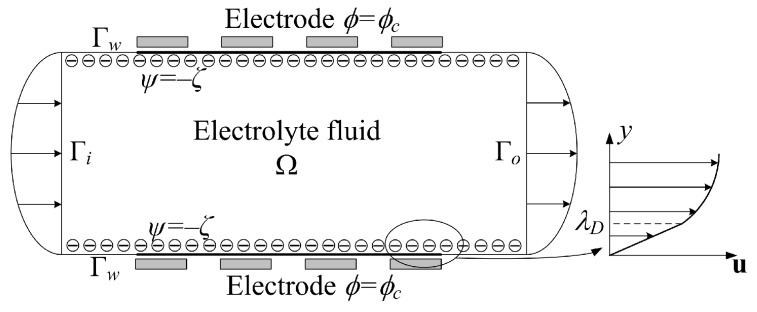
Schematic for the electroosmotic flow in the micromixer.

Under the precondition of the continuum assumption, the electroosmotic flow is described by the Navier–Stokes equations modified to include an electrical driving force term to represent the interaction between the excess ions of the electrical double layer (EDL) and the external electric field induced by the electrode potential, where an assumption is made that the Joule heating effect is negligible and can be ignored [32]. In electroosmotic flows, the electric potential can be decomposed into an external electric potential due to the imposition of the externally applied electrode potential and an electric potential due to surface wall charge [33]. Then, the body force imposed on the fluid is the electric force of these two potentials. Based on the above description, the governing equations of the electroosmotic flow are

(2) $$\begin{array}{c} \rho u\cdot \nabla u=\nabla \cdot \left[-pI+\eta \left(\nabla u+\nabla u^{T}\right)\right]+\frac{\varepsilon }{\lambda_{D}^{2}}\psi \nabla \phi ,\;\mathrm{in}\;\Omega \\ -\nabla \cdot u=0,\;\mathrm{in}\;\Omega \end{array}$$

where u is the fluid velocity; *p* is the fluid pressure; ρ and η are the density and viscosity of the fluid, respectively; $\lambda_{D}$ is the Debye length, which is the characteristic thickness of the EDL for a given solid–electrolyte liquid interface, and it is calculated to be $\lambda_{D}={\left(\varepsilon \varepsilon_{0}k_{b}T/2n^{0}z^{2}e^{2}\right)}^{1/2}$, with ε and $\varepsilon_{0}$ representing the dielectric constant of the electrolyte solution and free space, $k_{b}$ representing the Boltzmann constant, *T* representing the temperature, $n^{0}$ and *z* representing the concentration and valence of the ion in the electrolyte solution, and *e* representing the charge of the electron [12]; ψ is the electric potential due to surface wall charge; ϕ is the external electric potential; Ω is the space domain occupied by the electroosmotic flow, and the boundaries of Ω include the inlet port $\Gamma_{i}$, the outlet port $\Gamma_{o}$, and the sidewalls $\Gamma_{w}$ (Figure 1). The imposed boundary conditions for the Navier–Stokes equations are:

(3) $$\begin{array}{c} u=u_{i},\;\mathrm{on}\;\Gamma_{i}\\ u=0,\;\mathrm{on}\;\Gamma_{w}\\ \left[-pI+\eta \left(\nabla u+\nabla u^{T}\right)\right]\cdot n=0,\;\mathrm{on}\;\Gamma_{o} \end{array}$$

where $u_{i}$ is a given velocity distribution at the inlet port; n is the unit outward normal vector on the boundary of Ω. In micromixing, the two factors that influence the mixing performance of a micromixer are diffusion and chaotic advection. The mixing of flows is described using the convection–diffusion equation

(4) $$\begin{array}{c} u\cdot \nabla c=D\nabla^{2}c,\;\mathrm{in}\;\Omega \end{array}$$

where *D* is the diffusion constant of the fluid. The imposed boundary conditions for the convection–diffusion equation are:

(5) $$\begin{array}{c} c=c_{i}\left(x\right),\;\mathrm{on}\;\Gamma_{i}\\ \nabla c\cdot n=0,\;\mathrm{on}\;\Gamma_{w}\cup \Gamma_{o} \end{array}$$

where $c_{i}$ is the given concentration distribution at the inlet port of the electroosmotic micromixer. For a symmetrical and univalent electrolyte at room temperature, the Debye length is on the magnitude 10 nm for a concentration of $10^{-3}$ M. In other words, the Debye length is very small compared to the characteristic length of the microchannel [32]. Moreover, within the EDL, the electrical potential drops from the zeta potential to zero [12,32]. In general, the zeta potential is on the order of 0.1 V. The ion distribution in the EDL is influenced primarily by the zeta potential, and the distribution of the potential due to surface wall charge can be obtained by solving the equation

(6) $$\begin{array}{c} \nabla^{2}\psi =\frac{1}{\lambda_{D}^{2}}\psi ,\;\mathrm{in}\;\Omega \end{array}$$

For Equation (6), the imposed boundary conditions are

(7) $$\begin{array}{c} \psi =-\zeta ,\;\mathrm{on}\;\Gamma_{w}\\ \nabla \psi \cdot n=0,\;\mathrm{on}\;\Gamma_{i}\cup \Gamma_{o} \end{array}$$

where ζ is the zeta potential. Since the external electric potential arises from external charges, it satisfies a Laplacian equation within the fluid domain

(8) $$\begin{array}{c} \nabla^{2}\phi =0,\;\mathrm{in}\;\Omega \end{array}$$

and the corresponding boundary conditions are

(9) $$\begin{array}{c} \phi =\phi_{c}\left(x\right),\;\mathrm{on}\;\Gamma_{w}\\ \nabla \phi \cdot n=0,\;\mathrm{on}\;\Gamma_{i}\cup \Gamma_{o} \end{array}$$

where $\phi_{c}$ is the electrode potential on the sidewalls of the electroosmotic micromixer. Then, the micromixing in the electroosmotic flow can be described using the coupled system of Equations (2), (4), (6), and (8).

**Figure 2:**
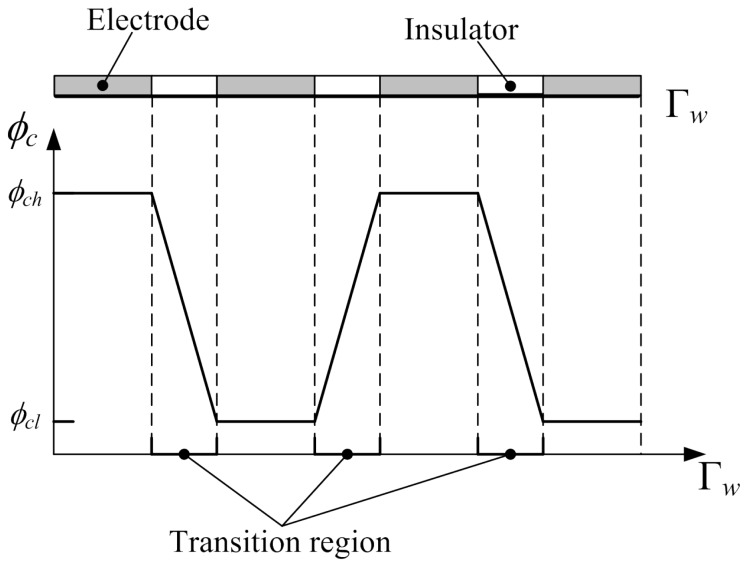
Schematic for the electrode and the corresponding external electric potential at the sidewall of the electroosmotic micromixer.

Based on the above description, the optimal control problem used to find the reasonable distribution of the electrode potential and minimize the mixing measurement can be constructed with the mixing measurement as objective, the coupling system of Equations (2), (4), (6), and (8) as constraints, and the electrode potential as control variable. Because the control variable (i.e., electrode potential) is defined on the sidewalls (the Dirichlet boundary of the coupled system), the constructed optimal control problem is a Dirichlet boundary control problem. In the optimal control problem, the admissable set of the control variable is set to be $\left[\phi_{cl},\phi_{ch}\right]$, where the values of $\phi_{cl}$ and $\phi_{ch}$ can be determined due to the engineering reality. In order to ensure the manufacturability of the obtained electrode distribution, the distribution of the electrode potential corresponding to the electrode distribution should satisfy the conditions as demonstrated in Figure 2: the electrode potential on every electrode should be an electric level corresponding to the constant potential $\phi_{cl}$ or $\phi_{ch}$; the size of the transition region—filled with insulators—between two neighboring electrodes should be large enough to avoid excess high electric field strength and capacitor breakdown. These conditions can be ensured using the filter and projection methods and by imposing a constraint on the electric field strength, where the control variable is filtered using the Helmholtz filter and the filtered variable is projected using the threshold method in this paper [34,35,36]. The control variable is evolved using the robust numerical optimization algorithm MMA (the method of moving asymptotes) [37,38]. Based on the filtering of the control variable, the reasonable distance between two neighboring electrodes at the sidewall can be ensured, and the filter is implemented by solving the following Helmholtz-type PDE:

(10) $$\begin{array}{c} -r^{2}\nabla_{\Gamma }^{2}{\tilde{\phi }}_{c}+{\tilde{\phi }}_{c}=\phi_{c},\;\mathrm{on}\;\Gamma_{w}\\ n_{\Gamma }\cdot \nabla_{\Gamma }{\tilde{\phi }}_{c}=0,\;\mathrm{at}\;\partial \Gamma_{w} \end{array}$$

where ${\tilde{\phi }}_{c}$ is the filtered control variable; *r* is the filter radius; $\nabla_{\Gamma }$ is the gradient operator defined on $\Gamma_{w}$; $n_{\Gamma }$ is the unit outward normal vector on the boundary $\Gamma_{w}$. The distance between two neighboring electrodes can be controlled by reasonably choosing the value of the filter radius to control the size of the transition region. Generally, a higher value of filter radius corresponds to a larger size of the transition region. The threshold projection can ensure that the change of the external electric potential is as linear as possible in the transition region between two neighboring electrodes on the sidewall, and it is performed using the following formulation:

(11) $$\begin{array}{c} {\bar{\tilde{\phi }}}_{c}=\frac{\tanh\left(\beta \xi \right)+\tanh\left(\beta \left({\tilde{\phi }}_{c}-\xi \right)\right)}{\tanh\left(\beta \xi \right)+\tanh\left(\beta \left(1-\xi \right)\right)} \end{array}$$

where ${\bar{\tilde{\phi }}}_{c}$ is the projected control variable; $\xi \in \left[0,1\right]$ and β are the threshold and projection parameters for the threshold projection, respectively. On the choice of the values of ξ and β, one can refer to [39]. Using the threshold projection, the filtered control variable can also be projected to $\phi_{cl}$ or $\phi_{ch}$ at the points in the region corresponding to the electrodes, and the interim values in $\left(\phi_{cl},\phi_{ch}\right)$ are avoided effectively; i.e., the external electric potential applied on the control boundary will only have the constant values $\phi_{cl}$ and $\phi_{ch}$, which can be realized by fabricating separated electrodes on the sidewall of the electroosmotic micromixer. To avoid excess high electric field strength, the electric field strength induced by the external electric potential is constrained as

(12) $$\int_{\Omega }{\left|\nabla \phi \right|}^{2}\;d\Omega \leq C_{0}$$

where $C_{0}$ is a constant, chosen based on numerical experiments and engineering reality. In Equation (12), $\int_{\Omega }{\left|\nabla \phi \right|}^{2}\;d\Omega$ is constrained to be less than an upper bound. In fact, the least upper bound $\sup_{\Omega }\left|\nabla \phi \right|$—the maximum of the electric field strength on Ω—should be constrained. Mathematically, $\sup_{\Omega }\left|\nabla \phi \right|$ and ${\left(\int_{\Omega }{\left|\nabla \phi \right|}^{2}\;d\Omega \right)}^{1/2}$ are equivalent, because they are respectively the *∞*- and 2-norm of $\left|\nabla \phi \right|$ on Ω. Therefore, the well-posed $\left(\int_{\Omega }{\left|\nabla \phi \right|}^{2}\;d\Omega \right)$ is used to impose the constraint for the upper bound of the electric field strength. Then, the manufacturability of the design corresponding to the result of the optimal control problem is ensured based on the Helmholtz filter, threshold projection, and electric field strength constraint. For summary, the optimal control problem for inverse determination of the electrode distribution for electroosmotic micromixer can be constructed to be:

(13) $$\begin{array}{c} \min\;\Psi \left(c\right)=\int_{\Gamma_{o}}{\left(c-c_{a}\right)}^{2}\;d\Gamma /\int_{\Gamma_{i}}{\left(c_{r}-c_{a}\right)}^{2}\;d\Gamma \\ s.t.\left\{\begin{array}{c} \rho u\cdot \nabla u=\nabla \cdot \left[-pI+\eta \left(\nabla u+\nabla u^{T}\right)\right]+\frac{\varepsilon }{\lambda_{D}^{2}}\psi \nabla \phi ,\;\mathrm{in}\;\Omega \\ -\nabla \cdot u=0,\;\mathrm{in}\;\Omega \\ u\cdot \nabla c=D\nabla^{2}c,\;\mathrm{in}\;\Omega \\ \nabla^{2}\psi =\frac{1}{\lambda_{D}^{2}}\psi ,\;\mathrm{in}\;\Omega \\ \int_{\Omega }{\left|\nabla \phi \right|}^{2}\;d\Omega \leq C_{0} \end{array}\right. \end{array}$$

By solving the optimal control problem, the electrode distribution corresponding to the external electric potential can be determined, and minimal of the mixing measurement can be derived.

### 2.2. Analyzing and Solving

The constructed optimal control problem in Section 2.1 is an optimization problem with partial differential equation constraints, and it can be analyzed using the adjoint method [40]. In this paper, the optimal control problem is solved by the finite element method. To use the linear elements for the partial differential equations, the Navier–Stokes equations and convection–diffusion equation are stabilized using the generalized least squares (GLS) and the streamline upwind Petrov–Galerkin (SUPG) technologies, respectively [41]. Then, the stabilized weak forms are

(14) $$\begin{array}{c} \int_{\Omega }\rho u\cdot \nabla u\cdot v+\int_{\Omega }\left[-pI+\eta \left(\nabla u+\nabla u^{T}\right)\right]:\nabla v\;d\Omega -\int_{\Omega }\frac{\varepsilon }{\lambda_{D}^{2}}\psi \nabla \phi \cdot v\;d\Omega -\int_{\Omega }q\nabla \cdot u\;d\Omega \\ +\sum_{i=1}^{N_{e}}\int_{\Omega_{i}}\tau_{GLS}\nabla p\cdot \nabla q\;d\Omega =0,\;\forall v\in H_{1}\left(\Omega \right),\;\forall q\in L_{2}\left(\Omega \right)\\ u=u_{i},\;\mathrm{on}\;\Gamma_{i}\\ u=0,\;\mathrm{on}\;\Gamma_{w} \end{array}$$

for the Navier–Stokes equations, and

(15) $$\begin{array}{c} \int_{\Omega }u\cdot \nabla c\;s\;d\Omega +\int_{\Omega }D\nabla c\cdot \nabla s\;d\Omega +\sum_{i=1}^{N_{e}}\int_{\Omega_{i}}\tau_{SUPG}u\cdot \nabla s\left(u\cdot \nabla c\right)\;d\Omega =0,\\ \forall s\in H_{1}\left(\Omega \right)\\ c=c_{i},\;\mathrm{on}\;\Gamma_{i} \end{array}$$

for the convection–diffusion equation, where $H_{1}\left(\Omega \right)$ and $L_{2}\left(\Omega \right)$ are the first-order Sobolev space and the second-order Lebesgue integrable functional space, respectively; $N_{e}$ is the number of elements used to discretize the computational domain; and $\Omega_{i}$ is the domain of the i-th element; $\tau_{GLS}$ and $\tau_{SUPG}$ are the stabilization parameters. The stabilization parameters are chosen according to [41,42]

(16) $$\begin{array}{c} \tau_{GLS}=\frac{h^{2}}{12\eta }\\ \tau_{SUPG}={\left(\frac{4}{h^{2}D}+\frac{2\left|u\right|}{h}\right)}^{-1} \end{array}$$

where *h* is the element size. Based on the adjoint analysis of the objective in Equation (1), the weak form of the adjoint equations of the convection–diffusion equation, the Navier–Stokes equations, and the Laplacian equation are obtained as: find $c_{a}\in H_{1}\left(\Omega \right)$, $u_{a}\in H_{1}\left(\Omega \right)$, $p_{a}\in L_{2}\left(\Omega \right)$, and $\phi_{a}\in H_{1}\left(\Omega \right)$ satisfying

(17) $$\begin{array}{c} \int_{\Omega }\left(u\cdot \nabla s\;c_{a}+\nabla s\cdot \nabla c_{a}\right)\;d\Omega +\sum_{i=1}^{N_{e}}\int_{\Omega_{i}}\tau_{SUPG}u\cdot \nabla c_{a}\left(u\cdot \nabla s\right)\;d\Omega \\ +\int_{\Gamma_{o}}2\left(c-c_{a}\right)s\;d\Gamma /\int_{\Gamma_{i}}{\left(c_{r}-c_{a}\right)}^{2}\;d\Gamma =0,\;\forall s\in H_{1}\left(\Omega \right)\\ c_{a}=0,\;\mathrm{on}\;\Gamma_{i} \end{array}$$

for the convection–diffusion equation, and

(18) $$\begin{array}{c} \int_{\Omega }\left\{\rho \left(v\cdot \nabla u+u\cdot \nabla v\right)\cdot u_{a}+\left[\eta \left(\nabla v+\nabla v^{T}\right)-qI\right]:\nabla u_{a}-p_{a}\nabla \cdot v\right\}\;d\Omega +\\ \sum_{i=1}^{N_{e}}\int_{\Omega_{i}}\tau_{GLS}\nabla q\cdot \nabla p_{a}\;d\Omega =-\int_{\Omega }v\cdot \nabla c\;c_{a}\;d\Omega -\\ \sum_{i=1}^{N_{e}}\int_{\Omega_{i}}[\left(\frac{\partial \tau_{SUPG}}{\partial u}\cdot v\right)\left(u\cdot \nabla c_{a}\right)\left(u\cdot \nabla c\right)\\ +\tau_{SUPG}v\cdot \nabla c_{a}\left(u\cdot \nabla c\right)+\tau_{SUPG}\left(u\cdot \nabla c_{a}\right)\left(v\cdot \nabla c\right)]\;d\Omega ,\;\forall v\in H_{1}\left(\Omega \right),\\ \forall q\in L_{2}\left(\Omega \right)\\ u_{a}=0,\;\mathrm{on}\;\Gamma_{i}\cup \Gamma_{w} \end{array}$$

for the Navier–Stokes equations, and

(19) $$\begin{array}{c} -\int_{\Omega }\nabla \phi_{a}\cdot \nabla \varphi \;d\Omega +\int_{\Omega }\frac{\varepsilon }{\lambda_{D}^{2}}\nabla \cdot \left(\psi u_{a}\right)\varphi \;d\Omega -\int_{\Gamma_{o}}\frac{\varepsilon }{\lambda_{D}^{2}}\psi u_{a}\cdot n\varphi \;d\Gamma =0,\;\forall \varphi \in H_{1}\left(\Omega \right)\\ \phi_{a}=0,\;\mathrm{on}\;\Gamma_{w} \end{array}$$

for the Laplacian equation, where $c_{a}$, $u_{a}$, $p_{a}$, and $\phi_{a}$ are the adjoint variables corresponding to *c*, u, *p*, and ϕ, respectively. In the adjoint analysis, Equation (6) need not be included, because the potential due to surface wall charge is independent of the externally applied potential. The adjoint sensitivity of the optimal control problem can be obtained as

(20) $$\begin{array}{c} \delta \hat{\Psi }=\int_{\Gamma_{w}}-\nabla \phi_{a}\cdot n\left(\phi_{ch}-\phi_{cl}\right)\frac{d{\bar{\tilde{\phi }}}_{c}}{d{\tilde{\phi }}_{c}}\frac{d{\tilde{\phi }}_{c}}{d\phi_{c}}\delta \phi_{c}\;d\Gamma \end{array}$$

For the constraint in Equation (12), the weak form of the adjoint equation is

(21) $$\begin{array}{c} \int_{\Omega }\nabla \left(\phi_{a}-\phi \right)\cdot \nabla \psi \;d\Omega =0,\;\forall \varphi \in H_{1}\left(\Omega \right)\\ \phi_{a}=0,\;\mathrm{on}\;\Gamma_{w} \end{array}$$

and the adjoint sensitivity is

(22) $$\delta C=\int_{\Gamma_{w}}\nabla \left(\phi -\phi_{a}\right)\cdot n\left(\phi_{ch}-\phi_{cl}\right)\frac{d{\bar{\tilde{\phi }}}_{c}}{d{\tilde{\phi }}_{c}}\frac{d{\tilde{\phi }}_{c}}{d\phi_{c}}\delta \phi_{c}\;d\Gamma$$

In the discretization of the sensitivities in Equations (20) and (22), $\frac{d{\tilde{\phi }}_{c}}{d\phi_{c}}$ should be treated skillfully to avoid the inverse of matrix; for details, one can refer to [34].

Solving of the optimal control problem is implemented using the gradient-based iterative approach listed in Table 1. In the iterative procedure, the coupled system of Equations (2), (4), (6), and (8), and the corresponding adjoint equations in the weak form are solved by the finite element method using the commercial software COMSOL Multiphysics (version 3.5) with linear elements (http://www.comsol.com). Then, the adjoint derivative can be obtained according to Equation (20). The discretized control variable is updated using MMA until the convergence criteria are satisfied, where the convergence criteria are set to be the maximal change of the control variable in consecutive 5 iterations less than $1\times 10^{-3}$ or the maximal iteration number 400.

## 3. Results and Discussion

**Figure 3:**
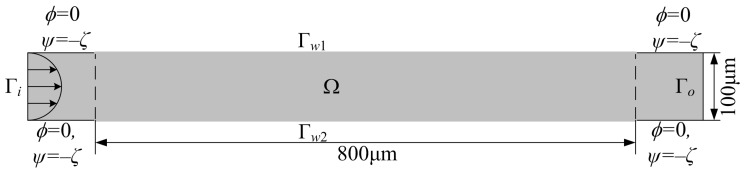
Schematic of the electroosmotic micromixer in a straight microchannel.

**Figure 4:**
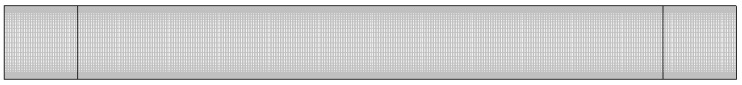
The mesh with rectangular elements used in the numerical computation, where the element size increases exponentially from the wall to the center of the channel. This mesh is fine enough to ensure the EDL is discretized by 10 elements in the scale of 10 nm.

To demonstrate the effectiveness of the proposed method used to inversly determine the electrode distribution for electroosmotic micromixers, an electroosmotic micromixer in a straight microchannel with externally applied electric potential imposed on the sidewalls is investigated numerically in the following. The schematic of the electroosmotic micromixer is shown in Figure 3, where the parabolic fluid velocity is loaded at the inlet $\Gamma_{i}$. In the numerical computation, the electroosmotic micromixer shown in Figure 3 is discretized using the mesh with rectangular elements shown in Figure 4, where the element size increases exponentially from the wall to the center of the channel. This mesh is fine enough to ensure that the EDL is discretized by 10 elements in the scale of 10 nm. The Reynolds number and Péclet number of the flow in the micromixer are 1 and 1000, respectively. The dielectric constant of the electrolyte solution, the Debye length, and the zeta potential are set to be $7.4\times 10^{-11}\;C^{2}/{\left(N\cdot m\right)}^{2}$, 765 nm, and 0.1 V, respectively. The bounds of the external electric potential are set as $\phi_{cl}=0$ V and $\phi_{ch}=200$ V. The upper bound of the constraint in Equation (12) is chosen to be $C_{0}=5.7\times 10^{5}$. Such choice of the parameter $C_{0}$ is to enforce that the externally applied electric field strength is no more than the general value $10^{7}$ V/m [22].

**Figure 5:**
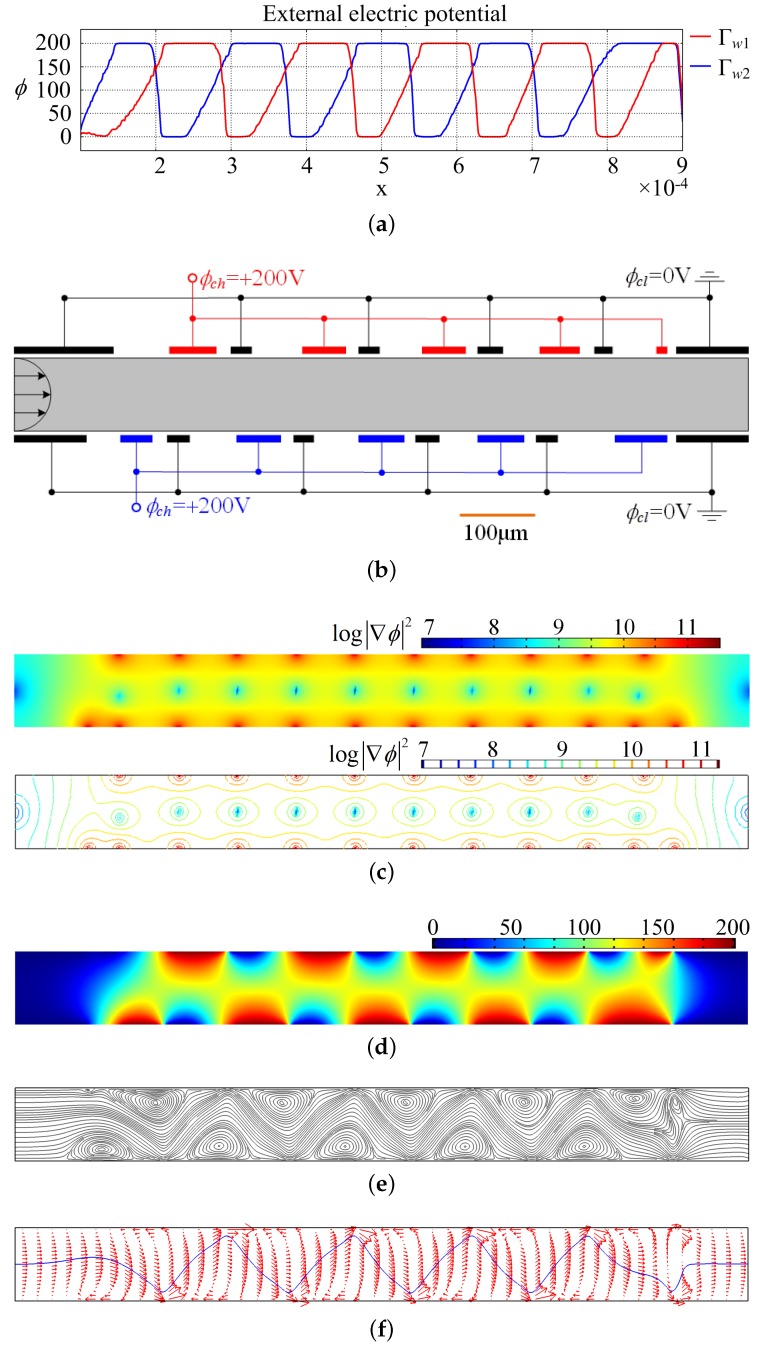
(**a**) Electrode potential obtained using the optimal control method; (**b**) electrode distribution corresponding to the obtained electrode potential; (**c**) logarithmic distribution and contours of the square of the electric field; (**d**) electric potential distribution induced by the obtained wall potential; (**e**) streamline distribution in the electroosmotic micromixer; (**f**) velocity distribution (red arrows) and anticipated concentration contour (blue curve) in the electroosmotic flow, where the chaotic advection and deformation of the interface between fluids is demonstrated.

**Figure 6:**
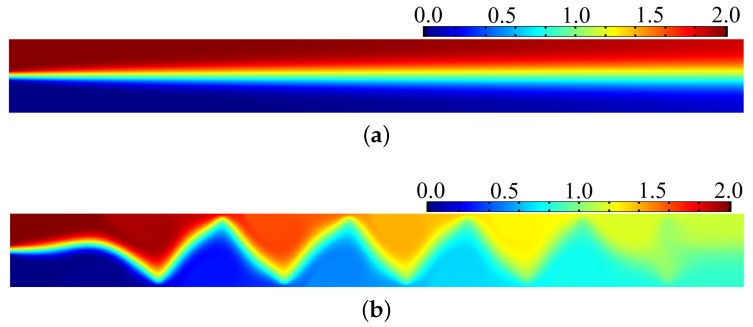
(**a**) Concentration distribution in the microchannel without electrode potential, where the value of the mixing measurement is 0.6; (**b**) concentration distribution with the electrode potential shown in Figure 5a, and the corresponding value of the mixing measurement is 0.015, which is lower than the threshold level of mixing, defined as 0.050 [8]. Therefore, complete mixing is achieved when the electrode potential obtained using optimal control method is imposed on the sidewalls of the electroosmotic micromixer in Figure 3.

Based on the optimal control theory in Section 2, the optimal distribution of the electrode potential is obtained as shown in Figure 5a. In Figure 5a, the low and high levels correspond to the electrodes with electric potentials equal to 0V and 200V, respectively; the declining parts between the neighboring low and high levels correspond to the regions filled with insulators used to separate neighboring electrodes. Therefore, the electrode distribution at the sidewalls of the electroosmotic micromixer can be determined according to the above analysis of the obtained optimal distribution of the externally applied electric potential (Figure 5b). Figure 5b shows that the electrodes have an interlaced arrangement. The interlaced arrangement of the low and high levels can effectively avoid the counteraction of the electric force loaded on the electrolyte; the electrodes are different sizes and the separation distances are different along the length of the microchannel; this is because two fluidic flows are mixed and the concentration distributions are different in different cross-sections of the microchannel. The distribution of the electric potential, induced by the electrode potential, is shown in Figure 5c. From Figure 5c, one can see that a high gradient of electric potential (electric strength) is produced near the region between neighboring electrodes. The high electric potential gradient results in the large electric force load on the electrolyte. Therefore, the streamlines of the microflow are distorted impetuously, and vortexes arise along with the distortion of the streamlines in the straight microchannel (Figure 5d). The distortion of the streamline and induced vortexes along the flow direction give rise to the enhancement of the chaotic advection, which is an interplay between the inertial, centrifugal, and viscous effects of the fluid flow. The enhancement of the chaotic advection strongly deforms the interface between fluids, the area of the interface grows exponentially, and diffusion becomes efficient (Figure 5e). As shown in Figure 5e, the electrical forces are loaded on the electrolyte in the electrical double layer, and this results in the reverse fluid velocity in the electrical double layer; furthermore, the reverse fluid velocity induces the chaotic advection of the flows in the micromixer. Therefore, the electrode distribution corresponding to the obtained electrode potential improves the micromixing effectively, and this can be confirmed based on the comparison between Figure 6a,b.

**Figure 7:**
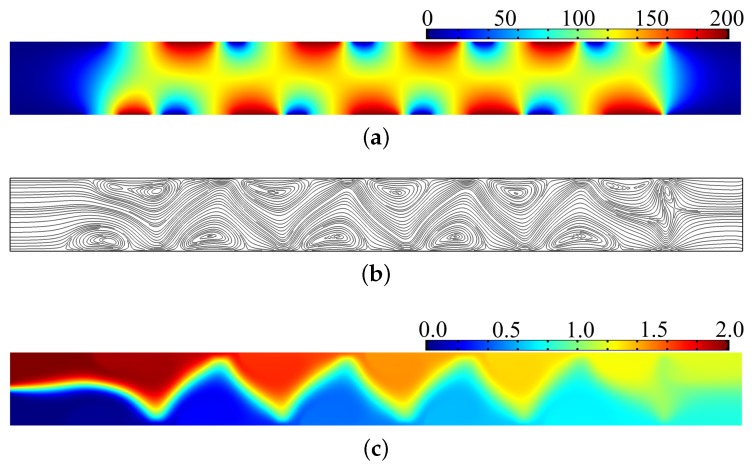
(**a**) Electric potential distribution corresponding to the electrode distribution shown in Figure 5b; (**b**) streamline distribution induced by the electrode distribution shown in Figure 5b; (**c**) concentration distribution in the electroosmotic micromixer with electrode distribution as shown in Figure 5b, and the value of the mixing measurement is 0.025 lower than the mixing threshold 0.050.

In the following, the postprocessing of the numerical results is performed. With the electrode distribution shown in Figure 5b, the distribution of the electric potential, streamline, and concentration are computed and shown in Figure 7. From the comparison between the results in Figure 5 and Figure 7, the consistency between the electric potential distributions corresponding to the optimal control method and the electrode distribution determined according to the electrode potential can be confirmed; and the effectiveness of the proposed method used to determine the electrode distribution for electroosmotic micromixers is demonstrated.

For manufacturability, the offset distance caused by fabrication tolerance will increase the distance between the electrodes on the two sides of the channel, when the electrodes are offset from the wall; sequentially, the electric field strength will be decreased and the electrical forces loaded on the electrolyte are weakened. Therefore, the mixing efficiency of the electroosmotic micromixer will be decreased when the electrodes are offset from the wall. In this paper, we considered the two-dimensional model. Therefore, the electrodes are assumed to cover the whole wall height. The case with the electrodes only covering a part of the wall height should be considered using the model in three dimensions. This will be implemented in our further and future research.

## 4. Conclusions

In this paper, the inverse method used to determine the electrode distribution for electroosmotic micromixers has been proposed based on the optimal control method. The electrode distribution is inversely determined based on solving one optimal control problem to minimize the mixing measurement. Additionally, the optimal control problem is constrained by the governing equations of the electroosmotic micromixing. The control variable is set to be the electrode potential distribution applied on the sidewall of the electroosmotic micromixer. The electric field strength in the micromixer has also been constrained to avoid the capacitor breakdown phenomenon. Based on the adjoint analysis of the optimal control problem, the control variable is evolved using MMA to derive potential distribution with low and high levels, which correspond to the electrode on the sidewall of the electroosmotic micromixer. The manufacturability of the obtained electrode distribution is ensured by the filtering and projection of the control variable. Numerical results demonstrated that the electrodes with an interlaced arrangement can effectively avoid the counteraction of the electric force loaded on the electrolyte; and the łeffectiveness of the proposed method is confirmed by the postprocessing of the numerical results. In addition, this method can be extended to determine the electrode distribution for the electroosmotic micromixers with unsteady flow caused by the AC electroosmosis. This will be investigated in future work.

## Figures and Tables

**Table 1:** Procedure of the iterative approach for solving the optimal control problem.

1. Give the initial value of the control variable $\phi_{c}$;
2. Solve the coupled system of Equations (2), (4), (6), and (8) by the finite element method;
3. Solve the weak form adjoint equations (Equations (17)–(19), and (21));
4. Compute the adjoint derivatives (Equations (20) and (22)) and
the corresponding objective and constraint values;
5. Update the control variable by MMA;
6. Check for convergence; if the stopping conditions are not satisfied, go to 2; and
7. Post-processing
